# Refinement of the extended crosswise model with a number sequence randomizer: Evidence from three different studies in the UK

**DOI:** 10.1371/journal.pone.0279741

**Published:** 2022-12-30

**Authors:** Khadiga H. A. Sayed, Maarten J. L. F. Cruyff, Peter G. M. van der Heijden, Andrea Petróczi

**Affiliations:** 1 Department of Methodology and Statistics, Utrecht University, Utrecht, The Netherlands; 2 Department of Statistics, Faculty of Economics and Political Science, Cairo University, Cairo, Egypt; 3 Department of Social Statistics and Demography, University of Southampton, Southampton, United Kingdom; 4 School of Life Sciences, Pharmacy and Chemistry, Kingston University, London, United Kingdom; 5 Department of Movement Sciences, KU Leuven, Leuven, Belgium; Saarland University, GERMANY

## Abstract

The Extended Crosswise Model (ECWM) is a randomized response model with neutral response categories, relatively simple instructions, and the availability of a goodness-of-fit test. This paper refines this model with a number sequence randomizer that virtually precludes the possibility to give evasive responses. The motivation for developing this model stems from a strategic priority of WADA (World Anti-Doping Agency) to monitor the prevalence of doping use by elite athletes. For this model we derived a maximum likelihood estimator that allows for binary logistic regression analysis. Three studies were conducted on online platforms with a total of over 6, 000 respondents; two on controlled substance use and one on compliance with COVID-19 regulations in the UK during the first lockdown. The results of these studies are promising. The goodness-of-fit tests showed little to no evidence for response biases, and the ECWM yielded higher prevalence estimates than direct questions for sensitive questions, and similar ones for non-sensitive questions. Furthermore, the randomizer with the shortest number sequences yielded the smallest response error rates on a control question with known prevalence.

## Introduction

Doping in elite sports has become a global challenge that threatens not only the fairness and justice of any sport competition, but also the health and careers of elite athletes [1]. Monitoring anti-doping activities worldwide and providing information on the prevalence of doping among elite athletes is one of the strategic priorities of the World Anti-Doping Agency (WADA). As custodian of the World-Anti-Doping Code 2021, WADA coordinates doping testing and publishes an annual list of prohibited substances and/or methods that defines doping in competitive sports [2]. Obtaining valid and reliable information on the prevalence of doping in competitive sports is essential to appraise the magnitude of the problem and to evaluate the effectiveness of global, national and regional anti-doping strategies [3]. Methods for the estimation of doping prevalence in elite sports include testing based on urine or blood analyses, the Athlete Biological Passport and survey methods [4]. A promising survey method for asking sensitive questions is using randomized response, because it is designed to eliminate evasive response biases by protecting the privacy of the respondents [5]. A meta-analysis has shown that randomized response designs provide more valid prevalence estimates than conventional direct questions (DQ) when questions are sensitive [6].

In 2011 WADA convened a working group to tackle the issue of doping prevalence and funded two studies for developing and testing a survey method for estimating doping prevalence at two international sport events: the 13^th^ International Association of Athletics Federations World Championships in Athletics (WCA) in Daegu and the 12^th^ Quadrennial Pan-Arab Games (PAG) in Doha [7]. The randomized response technique used in these surveys was the Unrelated Question Model (UQM) [8]. In the UQM, respondents are instructed to answer either a sensitive question or a non-sensitive (unrelated) question, depending on the outcome of a randomizer.

In [7], the UQM was implemented as follows. Respondents were instructed to think of the birthday of a person close to them, and to answer the unrelated question if the birthday fell in the first 10 days of the month, and to otherwise answer the question about doping use. The unrelated question was whether the birthday fell in the first six months of the year or not. The high prevalence estimates of 43.6% at WCA and 57.1% at PAG were unexpected, and raised doubts about the response validity. Several hypothesesabout instruction noncompliance were considered, but none of these fully explained the high estimates. To gain insight in the response validity in future applications, a refinement of the methodology was recommended.

The UQM has been employed in several studies to estimate the prevalence of various sensitive topics such as induced abortion, doping and illicit drug use in fitness sports and elite athletes, rape victimization and tax evasion. However, the “Yes” answer of this model is incriminating, and respondents may choose to provide a self-protective “No” response regardless of their true status to avoid being categorized as carriers of the sensitive characteristic [9]. Furthermore, its statistical model is saturated, which makes it impossible to test for the presence of response biases in the data.

**Fig 1:**
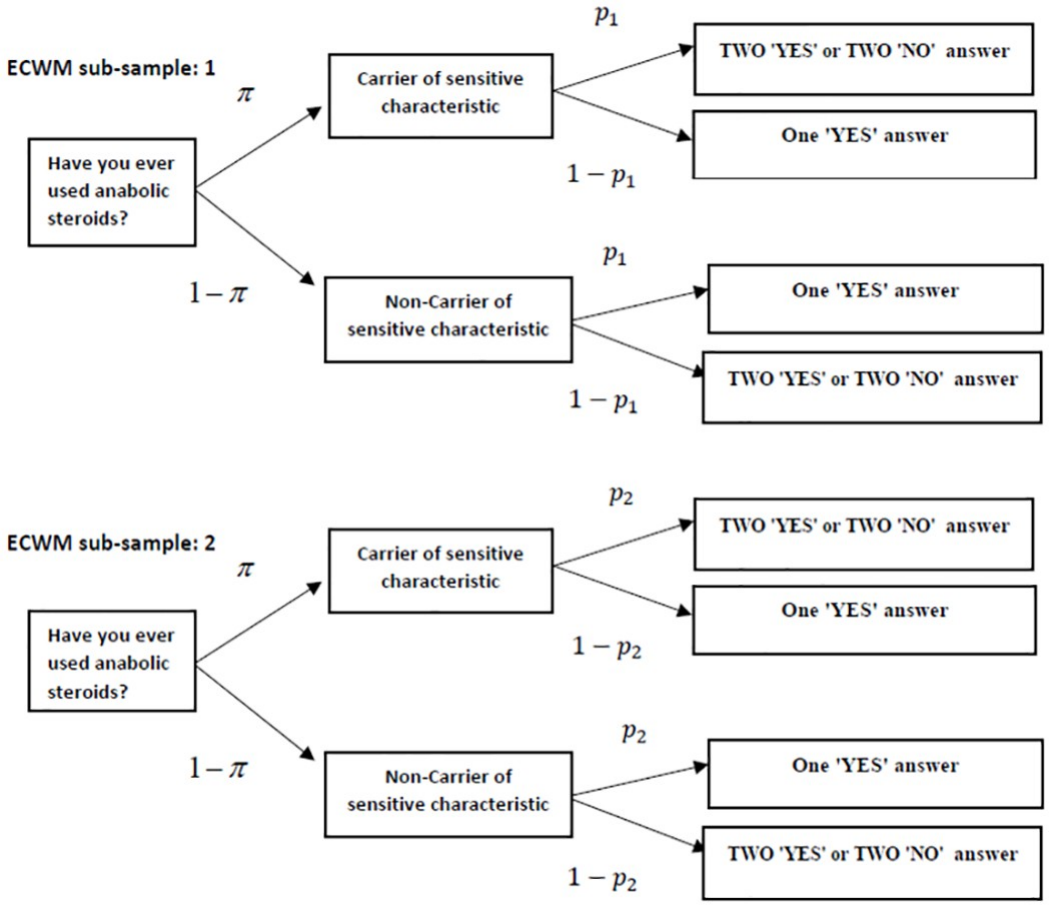
Diagram of the ECWM [26], with *π* denoting the prevalence of the sensitive characteristic, and *p*_1_ and *p*_2_, for *p*_1_ + *p*_2_ = 1, the probabilities of answering “Yes” to the unrelated question in sub-samples 1 and 2.

There are various indirect question techniques that avoid the use of incriminating responses, such as the Unmatched List technique [10], the Single Sample Count [11, 12], and the Crosswise Model (CWM) [13]. The CWM has gained sizable popularity since its inception in 2008, and thus offers ample empirical evidence for its performance in obtaining information on a range of socially sensitive issues [14–25]. Like the UQM, the CWM employs a sensitive and an unrelated question, but the respondent is instructed to answer both questions simultaneously with “I have TWO ‘Yes’ or TWO ‘No’ answers” or “I have ONE ‘Yes’ answer”. Since neither answer has an obvious incriminating connotation, both the urge and the potential for giving evasive responses is expected to be reduced. The statistical model, however, is still saturated and therefore unable to detect response biases. The Extended Crosswise Model (ECWM) extends the CWM by splitting the sample in two sub-samples, each with a different probability of answering “Yes” to the unrelated question. A diagram of the ECWM with two sub-samples is depicted in Fig 1, with *π* denoting the prevalence of the sensitive characteristic, and *p*_1_ and *p*_2_ the probability of answering “Yes” to the unrelated question in the subsequent sub-samples, for *p*_1_ = 1 − *p*_2_. The split in two samples with different randomization probabilities generates the necessary degree of freedom to conduct a goodness-of-fit test. Because of this property, and its neutral response categories, the ECWM was chosen. Since its introduction by [26], the ECWM has been applied in studies by [23, 27, 28].

**Fig 2:**
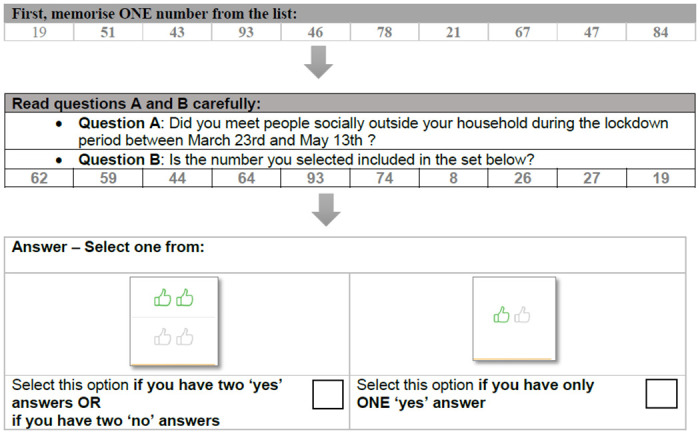
Example of the number sequence randomizer as used in Study II.

Addressing some of the concerns highlighted in the systematic review on CWM applications [29], we proposed a refinement of the ECWM with a number sequence randomizer rather than the birthday randomizer. A potential pitfall of the birthday randomizer is that birthdays may not be uniformly distributed over the months of the year, which in turn results in potentially biased probabilities to answer “Yes” to the unrelated question. This bias may be aggravated by the fact that in some countries birthdays are often unknown and arbitrarily set to January 1 (for an anecdotal example, see the Washington Post article “In Afghanistan, January 1 is everybody’s birthday” [30]). With the number sequence randomizer, the respondent is simply asked to memorize one number from a sequence of numbers, and the unrelated question is whether the memorized number is present or not in a second sequence of numbers. Since the probability that the memorized number reappears in the second sequence is exactly known, the potential biases of the birthday randomizer are avoided. The number sequence randomizer is discussed in detail in Section “Data and methods”, and for an example from Study II we refer to Fig 2. The ECWM by [26] in combination with our number sequence randomizer addresses these pitfalls.

The idea of splitting the sample in two sub-samples was originally proposed [31] in Cheater Detection Model (CDM) to estimate the prevalence of so-called ‘cheaters’, i.e. respondents who give the non-incriminating “No” answer when an incriminating “Yes” was required. Instead of cheaters we prefer the term ‘self-protective no-sayers’ as introduced by [32], because it provides a more accurate description of this type of respondents. The CDM has been used in studies to estimate the prevalence of doping, and yielded estimates of self-protective no-sayers ranging from 29.9% [33] to 22.9% and 30.6% [34]. The ECWM, however, is not capable of detecting self-protective no-saying. This is not as much because the answer “No” is not used, but because of the fact the incriminating response is not the same in both sub-samples. As can be inferred from Fig 1, carriers of the sensitive characteristic answer “I have TWO ‘Yes’ or TWO ‘No’ answers” with probability *p*_1_ in sub-sample 1, and with probability *p*_2_ in sub-sample 2, so that the self-protective responses are “I have One ‘Yes’ answer” in sub-sample 1, and “I have TWO ‘Yes’ or TWO ‘No’ answers” in sub-sample 2 assuming that *p*_1_ > *p*_2_. We call this kind of response behavior ‘informed self-protection’, because it requires an understanding of the connotation between the responses and the sensitive characteristic. As shown in Section “Statistical model”, the presence of informed self-protection cannot be detected because its effects on the observed response probabilities ‘cancel out’; as the fraction of misreported answers “I have TWO ‘Yes’ or TWO ‘No’ answers” in one sub-sample is expected to equal the fraction of misreported answers “I have ONE ‘Yes’ answer” in the other sub-sample. This does not render the goodness-of-fit test useless because it will be able to detect other response biases, as long as their effects on the observed response probabilities do not cancel out.

The aim of this study is to evaluate the performance of the ECWM in combination with the number sequence randomizer in three subsequent studies with socially sensitive behavioral choices to each; Study I and III concentrate on controlled substance use and Study II focuses on non-compliance with COVID-19 regulations. The performance of the ECWM is evaluated in several ways. Firstly, in line with the “more-is-better” criterion [6, 29, 35], we expect the ECWM to yield the same prevalence estimate as DQ when the question is non-sensitive, and higher estimates as the sensitivity of the question increases in the sense that the more sensitive the question, the more underreporting will occur when respondents are asked to answer the question directly. Secondly, we expect the goodness-of-fit tests for the ECWM questions to be non-significant. Additionally, we included a non-sensitive control question with a known prevalence of 100% to determine the effect of the length of the number sequences of our randomizer on the response error rate. Furthermore, by conducting logistic regressions analyses with the time spent to complete the survey and the perceived difficulty of the ECWM answer format we hope to gain some insight in the reasons for these response errors. Finally, we manipulated the format of the sensitive questions to investigate their effects on the prevalence estimates. In Study I we compare questions versus statements, and in Study II a factual versus judgmental questions. We hypothesized that the non-incriminating wording of the question in the factual format would encourage respondents to admit non-compliance with COVID-19 regulations, and thus yield higher prevalence estimates than the incriminating wording of the questions in the judgmental format.

The paper is structured as follows. In section “Statistical model”, we derive a maximum likelihood estimator for ECWM that also allows for the formulation of a regression model and for conducting the goodness-of-fit test. Section “Data and methods” provides information about participants, questionnaires, the procedure and measures of the three studies. Section “Results” evaluates the performance of the ECWM. Finally, the concluding remarks, limitations and directions for future research are presented in Sections “Discussion” and “Conclusion”.

## Statistical model

This section reviews the moment estimator of [26], and uses it to demonstrate that the ECWM is not capable of detecting informed cheating. Next a binary logistic regression model is derived, which is used to conduct the goodness-of-fit test.

### Moment estimation

A moment estimator is derived for the ECWM by [26] through fitting a separate model to each sub-sample separately. Let $\pi_{y|s}^{*}$ be the conditional probability of observing response *y*, for *y* ∈ {1 ≡ “I have ONE ‘Yes’ answer”, 2 ≡ “I have TWO ‘Yes’ or TWO ‘No’ answers”} given membership of sub-sample *s*, for *s* ∈ {1, 2}. Let *π*_*s*_ denote the prevalence of the sensitive characteristic in sub-sample *s*, and *p*_*s*_ the probability of a “Yes” answer to the unrelated question in sub-sample *s*, for *p*_1_ = 1 − *p*_2_ and *p*_1_ ≠ 0.5. The model for the ECWM is given by

$$\begin{array}{c} \pi_{y|s}^{*}=\{\begin{array}{c} p_{s}\pi_{s}+\left(1-p_{s}\right)\left(1-\pi_{s}\right)\;\text{for}\;y=2\\ \left(1-p_{s}\right)\pi_{s}+p_{s}\left(1-\pi_{s}\right)\;\text{for}\;y=1 \end{array}. \end{array}$$ (1)

Solving Eq (1) with respect to *π*_*s*_ for each sub-sample separately yields the moment estimators

$$\begin{array}{c} {\hat{\pi }}_{s}=\frac{{\hat{\pi }}_{2|s}^{*}-1+p_{s}}{2p_{s}-1};\;\;p_{s}\neq 0.5, \end{array}$$ (2)

where ${\hat{\pi }}_{2|s}^{*}$ is estimated by the conditional proportion *n*_2*s*_/*n*_*s*_ of “I have TWO ‘Yes’ or TWO ‘No’ answers” within sub-sample *s*. Note that the moment estimator Eq (2) yields a negative prevalence estimate ${\hat{\pi }}_{s}$ when in sub-sample *s* the numerator and denominator differ in sign. For example, this would happen when *p*_1_ = 0.8 and the proportion of “I have TWO ‘Yes’ or TWO ‘No’ answers” in sub-sample 1 is below 20%, so that ${\hat{\pi }}_{2|1}^{*}<0.2$.

Since under random assignment to the sub-samples $E\left({\hat{\pi }}_{1}\right)=E\left({\hat{\pi }}_{2}\right)=\pi$, the overall prevalence estimate is obtained as the weighted average

$$\begin{array}{c} \hat{\pi }=\frac{n_{1}}{n}{\hat{\pi }}_{1}+\frac{n_{2}}{n}{\hat{\pi }}_{2}, \end{array}$$ (3)

where *n*_1_ and *n*_2_ are the respective sub-sample sizes, and *n* = *n*_1_ + *n*_2_ the total sample size. Substituting 1 − *p*_1_ for *p*_2_, the variance of $\hat{\pi }$ is given by

$$\begin{array}{c} V(\hat{\pi })=\frac{1}{n}[\pi (1-\pi )+\frac{p_{1}(1-p_{1})}{{\left(2p_{1}-1\right)}^{2}}], \end{array}$$ (4)

which is identical to the variance of $\hat{\pi }$ under the CWM.

We now present an alternative moment estimator that estimates *π* directly instead estimating *π*_1_ and *π*_2_. In matrix notation the model is given by

$$\begin{array}{c} \pi_{y|s}^{*}=P\pi , \end{array}$$ (5)

where $\pi_{y|s}^{*}={\left(\pi_{2|1}^{*},\pi_{1|1}^{*},\pi_{2|2}^{*},\pi_{1|2}^{*}\right)}^{\prime }$, ***π*** = (*π*, 1 − *π*)′, and

$$\begin{array}{c} P=(\begin{array}{cc} p_{1} & 1-p_{1}\\ 1-p_{1} & p_{1}\\ p_{2} & 1-p_{2}\\ 1-p_{2} & p_{2} \end{array})=(\begin{array}{cc} p_{1} & 1-p_{1}\\ 1-p_{1} & p_{1}\\ 1-p_{1} & p_{1}\\ p_{1} & 1-p_{1} \end{array}), \end{array}$$ (6)

is a 4 × 2 transition matrix with the randomization probabilities of both sub-samples. In the right-hand side of Eq (6) we have substituted 1 − *p*_1_ for *p*_2_.

The moment estimator of *π* is then given by the single expression

$$\begin{array}{c} \hat{\pi }=2P^{-1}{\hat{\pi }}_{ys}^{*}, \end{array}$$ (7)

where ***P***^**−1**^ is the Moore-Penrose generalized inverse of ***P***, and ${\hat{\pi }}_{ys}^{*}$ the vector with the unconditional response probabilities as estimated by *n*_*ys*_/*n*. The estimated variance of $\hat{\pi }$ is given by

$$\begin{array}{c} \hat{V}\left(\hat{\pi }\right)=4n^{-1}P^{-1}\;(Diag\left({\hat{\pi }}_{ys}^{*}\right)-{\hat{\pi }}_{ys}^{*}{\hat{\pi }}_{ys}^{*}{}^{t})\left(P^{-1}\right){}^{t}. \end{array}$$ (8)

### Detection of informed self-protection

We now show that the statistical model of the ECWM accounting for informed self-protection is not identified. Let us first consider the situation without informed cheating under model Eq (5). From the equality of the rows 1 and 4 and rows 2 and 3 of the matrix ***P*** in Eq (6), it follows that $\pi_{2|1}^{*}=\pi_{1|2}^{*}$ and $\pi_{1|1}^{*}=\pi_{2|2}^{*}$. In the absence of response biases, we therefore expect to observe the equality of the response proportions ${\hat{\pi }}_{2|1}^{*}={\hat{\pi }}_{1|2}^{*}$ and ${\hat{\pi }}_{1|1}^{*}={\hat{\pi }}_{2|2}^{*}$ in our sample.

Now assume that carriers and non-carriers give informed self-protective answers with probabilities *θ*_*c*_ and *θ*_*nc*_. For *p*_1_ > *p*_2_, the sensitive response in sub-sample 1 is *y* = 2, so that here the respective probabilities *p*_1_ and 1 − *p*_1_ that carriers and non-carriers answer *y* = 2 are reduced by the respective factors 1 − *θ*_*c*_ and 1 − *θ*_*nc*_. In sub-sample 2 where *y* = 1 is the sensitive response, the effect of informed self-protection is in the opposite direction. This situation is described by the model ${\tilde{\pi }}_{y|s}^{*}=Q\pi$, where

$$\begin{array}{c} Q=(\begin{array}{cc} p_{1}\left(1-\theta_{c}\right) & \left(1-\theta_{nc}\right)\left(1-p_{1}\right)\\ 1-p_{1}\left(1+\theta_{c}\right) & p_{1}+\theta_{nc}\left(1-p_{1}\right)\\ 1-p_{1}\left(1+\theta_{c}\right) & p_{1}+\theta_{nc}\left(1-p_{1}\right)\\ p_{1}\left(1-\theta_{c}\right) & \left(1-\theta_{nc}\right)\left(1-p_{1}\right) \end{array}), \end{array}$$ (9)

is the transition matrix accounting for informed self-protection, and ${\tilde{\pi }}_{y|s}^{*}$ the vector with the conditional response probabilities. It is obvious that ${\tilde{\pi }}_{y|s}^{*}\neq \pi_{y|s}^{*}$ for *θ*_*c*_ > 0 and/or *θ*_*nc*_ > 0. The equality relations ${\tilde{\pi }}_{2|1}^{*}={\tilde{\pi }}_{1|2}^{*}$ and ${\tilde{\pi }}_{1|1}^{*}={\tilde{\pi }}_{2|2}^{*}$ however are not affected by informed self-protection, since ***Q*** has the same equality of the rows 1 and 4 and the rows 2 and 3 as ***P***. It follows that ${\tilde{\pi }}_{y|s}^{*}$ can also be explained by model ${\tilde{\pi }}_{y|s}^{*}=P\pi$, and that therefore both the parameters *θ*_*c*_ and *θ*_*nc*_ of the model ${\tilde{\pi }}_{y|s}^{*}=Q\pi$ are unidentified. And since ***P*** ≠ ***Q***, the estimator $\hat{\pi }$ of model (5) is biased in the presence of informed self-protection.

### Maximum likelihood estimation

In this section we derive a maximum likelihood estimator (MLE) for the ECWM. This MLE can be used to obtain a prevalence estimate of the sensitive characteristic, but can also be used to estimate the parameters of a logistic regression model [36, 37]. A goodness-of-fit test is performed by comparing the log likelihoods of the intercept-only model and the model with a covariate denoting sub-sample membership.

Let $\pi_{y_{is}}^{*}$ denote the probability of observing response *y* by individual *i* in sub-sample *s*, for *i* ∈ {1, …, *n*}, so that

$$\begin{array}{c} \pi_{y_{is}}^{*}=\{\begin{array}{c} p_{s}\pi_{i}+\left(1-p_{s}\right)\left(1-\pi_{i}\right)\;\text{for}\;y_{is}=2\\ \left(1-p_{s}\right)\pi_{i}+p_{s}\left(1-\pi_{i}\right)\;\text{for}\;y_{is}=1 \end{array}. \end{array}$$ (10)

Model (10) is converted into a logistic regression model by the specification of the logistic function

$$\begin{array}{c} \pi_{i}=\frac{\text{exp}(x_{i}^{\prime }\beta )}{1+\text{exp}(x_{i}^{\prime }\beta )}, \end{array}$$ (11)

where ***x***_*i*_ = (1, *x*_*i*1_, …, *x*_*ik*_)′ is the vector with covariates, and ***β*** = (*β*_0_, *β*_1_, …, *β*_*k*_)′ the corresponding vector with regression coefficients. The model is estimated by maximization of the log likelihood function

$$\begin{array}{c} \text{ln}\;\ell \left(\beta |{y,x}_{i}\right)=\sum_{i=1}^{n}\text{ln}\;\pi_{y_{is}}^{*}. \end{array}$$ (12)

The variance of the elements in $\hat{\beta }$ are given by the diagonal elements of the inverse of the Hessian matrix. These variances are on the logit scale, but if so desired they can be transformed to the probability scale by use of the delta method.

The estimate of the population prevalence *π* is obtained by fitting the intercept-only model, and the point and interval estimates of the moment and maximum likelihood estimators are identical as long as the moment estimators are non-negative prevalence estimates [38]. The inclusion of a covariate denoting sub-sample membership yields the same prevalence estimates ${\hat{\pi }}_{1}$ and ${\hat{\pi }}_{2}$ as [26] moment estimator (2). The goodness-of-fit test statistic (likelihood ratio statistic) is then computed as twice the difference of log likelihood of the models with and without the sub-sample membership covariate, which is identical to the *G*^2^ statistic given by $2\sum_{y}\sum_{s}n_{ys}\;\text{log}\left(n_{ys}/{\hat{n}}_{ys}\right)$, where *n*_*ys*_ are the observed response frequencies, and ${\hat{n}}_{ys}$ the fitted ones, unless the prevalence estimate is on the boundary of the parameter space (0, 1). If a boundary solution exists, the likelihood ratio statistic will be zero whereas the value of *G*^2^ statistic will be greater than zero. The likelihood ratio statistic has an approximate chi-squared distribution on 1 degree of freedom, and a significant value provides evidence for instruction non-adherence.

The R package ECWM for moment and maximum likelihood estimation of the ECWM is available at the github page https://github.com/Khadiga-S/ecwm.

## Data and methods

To evaluate the performance of the ECWM with the number sequence randomizer, three independent studies in non-athlete populations were conducted in 2020. Study II measures compliance with the UK Covid-19 lockdown rules between March 23^rd^ and May 13^th^, 2020. Study I and Study III measure the respondents’ experiences with dietary and herbal supplements, controlled stimulants and controlled drugs for performance and/or image enhancement. For a summary of the survey design and participants allocation to different survey formats in the three studies, see S1 Table.

### Participants

The participants in the three studies were recruited via the Prolific Academic Platform. Prolific is an online research platform that enables researchers to recruit participants for research online in a cost-effective and reliable way. Participants are paid for their contribution at or above the minimum living wage, pro rata for the time estimated for survey completion. There is some evidence that the Prolific participants produce data quality higher than other online platforms [39]. Prior to their participation, all respondents consented to participate in our study and written informed consent was obtained. The studies did not involve minors and were subject to full ethical review prior to the data collection from which we obtained favorable ethical opinions. Ethical approval to perform the three studies was obtained from the Research Ethics Committee of the Faculty of Science, Engineering and Computing, Kingston University London. Ethical approval for the data analysis was also obtained from Utrecht University.

The target respondents in all studies were English-speaking people over the age of 18 years. Study II was restricted for those who were in the UK during the lockdown period. Of 2,424 participants who started study II, 6 (0.25%) persons were not in the UK during the lockdown and another 16 (0.62%) did not complete the survey. In Study I, the survey started with 1,518 individuals, 13 (0.85%) did not complete the questionnaire and answered only the demographic questions, while of 1,811 participating in Study III, 10 (0.55%) did not complete the survey. The Prolific platform was set to prevent the same person participating in the other two studies. The final samples consist of 1,505 respondents in Study I, 2,402 study II and 1,801 study III. Additional information about some background characteristics of the participants in the three studies and power curves to determine minimum sample size required for each CWM condition, can be found in S1 Appendix and S1 Fig, respectively.

### Questionnaires

The questionnaires of the three studies began with a brief introduction explaining the purpose and content of the survey. Participants in Study II were informed that they would be asked four questions related to their daily routines during the COVID19 lockdown, whereas in Study I and Study III they would be asked four questions about their experiences with dietary and herbal supplements, controlled stimulants and controlled drugs for performance- and/or image-enhancement, as well as some demographic questions. They were informed that the survey would take less than 5 minutes to complete, and that they could withdraw from the study at any time if they are unable or unwilling to complete the survey. Participants were informed that voluntary completion of the survey constitutes informed consent to participate in the study, and assured that the survey is completely anonymous, so it is totally safe to be honest and answer truthfully.

### Procedures and measures

In each study, the survey began with a set of basic demographic questions about age, gender, highest level of education and the level of sport activity. Before progressing to the sensitive questions section, participants were asked to indicate their birth month or to select a month at random if they wish. It was explained to the participants that this information is only used to assign them to one of the experimental conditions with the same content, but without giving details on how this assignment was operationalized. Participants were blinded to the experimental condition allocation, which is detailed in S2 Fig. In Study II and Study III the participants were randomly assigned to either a DQ condition or ECWM condition, whereas in Study I the DQ condition was not employed. In Study I, the sensitive behaviour was presented as question or statement, while only the question format was applied in Study III. In Study II, the questions were presented in two different formats, namely Factual and Judgmental. Table 1 shows the observed response frequencies of the three studies in the DQ and the ECWM conditions. Participants in the DQ condition had to answer the sensitive questions with “Yes” or “No”, whereas in the ECWM condition they were asked to answer either “I have only one ‘Yes’ answer” without revealing which question was answered with “Yes”, or “I have two ‘Yes’ answers or none” without revealing whether it was two or none. The probabilities 1/5 and 4/5 of answering “Yes” to the unrelated question in the two ECWM conditions are indicated in brackets, and the answers “I have TWO ‘Yes’ or TWO ‘No’ answers” and “I have ONE ‘Yes’ answer” are denoted by 2 and 1, respectively. Respondents in the ECWM condition were asked an additional question about the perceived difficulty of the instructions. This question “How easy or difficult was it for you to answer this question format?” was scored on an easy to difficult scale from 0–100.

**Table 1:** Observed response frequencies of Studies I, II, and III.

	Experimental Conditions					
	DQ		ECWM (1/5)		ECWM (4/5)	
	Yes	No	2/0	1/1	2/0	1/1
**Study I-Question**						
**Drug use1-Q1**: Have you ever been drug tested, for any reason? (including breath alcohol tests)	-	-	249	112	150	223
**Drug use1-Q2**: Have you ever used nutritional or herbal supplements for reasons other than health? (for example to make you more alert, help with weight loss)	-	-	188	185	183	178
**Drug use1-Q3**: Have you ever used controlled psychoactive drugs? (e.g., cannabis, cocaine, ecstasy)	-	-	197	164	174	199
**Drug use1-Q4**: Have you ever used controlled sport drugs without medical need to enhance your athletic performance? (e.g., anabolic steroids, hormone)	-	-	275	98	120	241
**Study I-Statement**						
**Drug use1-S1**: I have been drug tested, at least once, in my life (including breath alcohol tests)	-	-	233	143	166	229
**Drug use1-S2**: I have used, at least once, nutritional or herbal supplements for reasons other than health (for example to make you more alert, help with weight loss)	-	-	197	198	171	205
**Drug use1-S3**: I have used, at least once, controlled psychoactive drugs (e.g., cannabis, cocaine, ecstasy)	-	-	187	189	208	187
**Drug use1-S4**: I have used controlled sport drugs without medical need, at least once, to enhance my athletic performance? (e.g., anabolic steroids, hormone)	-	-	304	91	120	256
**Study II-Factual**						
**Covid-F1**: Did you meet people socially outside your household during the lockdown period?	37	341	249	145	186	247
**Covid-F2**: Did you go out for shopping, errands or work more than it was necessary during the lockdown period	52	326	284	149	164	230
**Covid-F3**: Did you spend more than one hour outside your house exercising during the lockdown period?	128	250	233	161	181	252
**Covid-F4**: Did you use any form of transportation (including driving or cycling) to go for exercise outside your area during the lockdown period?	65	313	271	162	146	248
**Study II-Judgmental**						
**Covid-J1**: Did you break the social distancing rule during the lockdown period?	44	359	262	158	149	225
**Covid-J2**: Did you break the stay home rule during the lockdown period?	53	350	233	141	154	266
**Covid-J3**: Did you break the one-hour rule for outside exercise during the lockdown period?	103	300	237	183	151	223
**Covid-J4**: Did you break the stay in your area rule during the lockdown period?	28	375	210	164	155	265
**Study III-Question**						
**Drug use2-Q1**: Have you ever been drug tested, for any reason? (including breath alcohol tests)	121	469	438	163	232	378
**Drug use2-Q2**: Have you ever used nutritional or herbal supplements for reasons other than health? (for example to make you more alert, help with weight loss)	274	316	330	280	285	316
**Drug use2-Q3**: Have you ever used controlled psychoactive drugs? (e.g., cannabis, cocaine, ecstasy)	230	360	302	299	300	310
**Drug use2-Q4**: Have you ever used controlled sport drugs without medical need to enhance your athletic performance? (e.g., anabolic steroids, hormone)	26	564	478	132	138	463

The surveys were hosted on a closed survey platform (Surveymonkey) in all three studies. The number of eligible participants were 35,852 for Study I, 58,744 for Study II, and 28,368 for Study III. Data were collected on February 21^st^, May 22^nd^, and July 7^th^ 2020, respectively. On average, participants were paid at the level of £11.57/hr, £9.87/hr £16.88/hr taking part in Studies I, II, and III, respectively.

### The number sequence randomizer

In Studies I, II and III number sequence randomizers were used with respectively 15, 10 and 5 numbers. The first sequence consisted of randomly generated two-digit numbers, and the respondents were asked to memorize one of the numbers. In order to avoid selection bias, ‘lucky’ or easy-to-memorize numbers, the single-digit numbers 1 to 9, master numbers (11, 22, 33, 44, …) and multiples of ten (10, 20, 30, 40, …) were excluded from this sequence. Then a second number sequence of the same length was shown that potentially included all numbers from 1 to 99. Depending on the length of the first sequence and the sub-sample, 3, 2 or 1 numbers or 12, 8, or 4 numbers reappeared in the second sequence, so that the probability that the memorized number reappeared was 1/5 in one sub-sample and 4/5 in the other. These ratios were alternated between questions within each sub-sample. The respondents then had to answer the unrelated question “Is the number you selected included in the set below?” and the sensitive question. This procedure is illustrated in Fig 2 for the Covid-F1 question of Study II. In this example, two of the ten numbers from the first sequence reappear in the second, so in this example the probability of answering “Yes” to the unrelated question B is 1/5.

Since the first and second number sequence are not shown simultaneously, it makes it extremely difficult for the respondent to remember and keep track of how many numbers of the first sequence reappeared in the second one. Consequently, it is practically impossible for the respondent to infer the probability of a “Yes” answer to the unrelated question, and thus to figure out which is the incriminating response. Obviously, this virtually rules out the possibility of informed self-protection.

### Control question

Prolific participants receive payment for completing a survey, thus the more surveys they fill out, the higher their earnings. The reference to “earn more” means that the faster they complete a survey the better their hourly rate of pay is in the sense that the more time they have to participate in other surveys, posted by other researchers, completely independent of our study. Consequently, there is a risk of some form of survey ‘fatigue’ and/or ‘rush’, which may lead them to skip instructions or to careless answering of the questions. To assess the quality of respondents’ answers, the questionnaires of all studies included a control question with known prevalence, namely whether the participants were paid or not for participating in the survey. In the DQ condition this question has an expected prevalence estimate of 100%. To ensure the same expectation in the ECWM condition, the probability of answering “Yes” to the corresponding unrelated question in Study I and Study II was fixed at 1 by letting all numbers of the first sequence reappear in a different order in the second one. This probability was set to be zero in Study III. Therefore, in the ECWM condition we expect to see 100% “I have TWO ‘Yes’ or TWO ‘No’ answers” in the first two studies and 100% “I have ONE ‘Yes’ answer” in Study III. This setup allows us to compare the error rates under DQ and the ECWM. In both conditions the response errors are attributable to survey fatigue, but in the ECWM condition there is the additional risk of non-adherence due to misunderstanding of the instructions or recall errors when using the number sequence randomizer. By comparing the error rates under DQ and the EWCM, we may get an estimate of the fraction of the error rate that is specifically attributable to the ECWM. Furthermore, by regressing the answers to the control question on variables assessing the time spent on the survey and the perceived difficulty of the ECWM instructions, we hope to gain some insight in the reasons for these response errors.

## Results

This section presents the maximum likelihood prevalence estimates and goodness-of-fit statistics of all studies, followed by an analysis of the control question.

### Prevalence estimates and goodness-of-fit

Table 2 presents the prevalence estimates and goodness-of-fit statistics of the three studies. The last three columns present the difference of the prevalence estimates $\Delta \hat{\pi }={\hat{\pi }}_{ECWM}-{\hat{\pi }}_{DQ}$ between the ECWM and the DQ for each question separately, the values of test statistic $z=\Delta \hat{\pi }/\sqrt{var(\Delta \hat{\pi })}$, with $\text{var}\left(\Delta \hat{\pi }\right)=var\left({\hat{\pi }}_{ECWM}\right)+var\left({\hat{\pi }}_{DQ}\right)$, and the associated *p*-values. The results for Studies II and III with a DQ condition show that the prevalence estimates are in line with the “more-is-better” criterion; the ECWM yields significantly higher prevalence estimates than DQ when the questions are sensitive, and similar estimates when the questions are non-sensitive. The exceptions are Covid-F3, were the difference is not significant, and Drug use2-Q4, were DQ yields a (not significantly) higher prevalence estimate. Six goodness-of-fit tests are significant and show a lack of fit; three in Study I, two in Study II and one in Study III. Three of these only exceed the critical value of 3.84 by a small margin, and the largest *G*^2^ statistics occur on the non-sensitive Drug use-Q1 questions, which suggests that the lack of fit is not due to self-protective response biases, but to a misunderstanding of the instructions on the first question.

**Table 2:** Prevalence estimates of Studies I, II, and III.

	DQ $\hat{\pi }$ (95% CI)	ECWM			ECWM—DQ		
		$\hat{\pi }$ (95% CI)	$G_{\left(1\right)}^{2}$	*p*-value	$\Delta \hat{\pi }$	*z*-value	*p*-value
**Study I-Question**							
Drug use1-Q1 *	-	26.2 (20.4, 32.0)	6.77	0.009	-	-	
Drug use1-Q2 *	-	50.2 (44.2, 56.3)	0.09	0.767	-	-	
Drug use1-Q3	-	43.4 (37.4, 49.4)	0.11	0.740	-	-	
Drug use1-Q4	-	16.2 (10.7, 21.7)	4.27	0.039	-	-	
**Study I-Statement**							
Drug use1-S1 *	-	33.5 (27.7, 39.2)	1.28	0.258	-	-	
Drug use1-S2 *	-	46.4 (40.6, 52.3)	1.67	0.197	-	-	
Drug use1-S3	-	52.5 (46.6, 58.4)	0.44	0.507	-	-	
Drug use1-S4	-	12.3 (7.0, 17.5)	7.65	0.006	-	-	
**Study II-Factual**							
Covid-F1	9.8 (6.8, 12.8)	33.4 (27.8, 38.9)	3.26	0.071	23.6	7.31	0.000
Covid-F2	13.8 (10.3, 17.2)	29.7 (24.2, 35.3)	4.56	0.033	16.0	4.80	0.000
Covid-F3	33.9 (29.1, 38.6)	35.6 (30.0, 41.2)	0.07	0.784	1.7	0.45	0.652
Covid-F4	17.2 (13.4, 21.0)	28.7 (23.2, 34.2)	0.01	0.915	11.5	3.39	0.001
**Study II-Judgmental**							
Covid-J1	10.9 (7.9, 14.0)	31.1 (25.5, 36.8)	0.41	0.521	20.3	6.17	0.000
Covid-J2	13.2 (9.9, 16.5)	28.6 (23.0, 34.2)	0.09	0.764	15.5	4.64	0.000
Covid-J3	25.6 (21.3, 29.8)	36.8 (31.1, 42.5)	0.83	0.362	11.3	3.07	0.002
Covid-J4	6.9 (4.5, 9.4)	33.6 (27.9, 39.3)	3.97	0.046	26.9	8.45	0.000
**Study III-Question**							
Drug use2-Q1 *	20.5 (17.3, 23.8)	21.0 (16.6, 25.4)	16.46	0.000	0.5	0.19	0.850
Drug use2-Q2 *	46.4 (42.4, 50.5)	44.4 (39.7, 49.1)	0.28	0.596	-2.0	-0.63	0.531
Drug use2-Q3	39.0 (35.1, 42.9)	49.1 (44.4, 53.8)	0.04	0.843	10.1	3.23	0.001
Drug use2-Q4	4.4 (2.8, 6.1)	3.8 (0.0, 7.7)	0.31	0.580	-0.6	-0.27	0.791

* Non-sensitive question.

Table 3 presents the difference (*diff*.) of the ECWM prevalence estimates between the question and statement conditions of Study I, and the factual and judgmental conditions of Study II. The *p*-values of the test statistic *z* show that there are no significant differences between the corresponding questions, and in contrast to our expectation the factual condition did not yield consistently higher prevalence estimates than the judgmental condition.

**Table 3:** Comparison between question formats across ECWM condition.

	*diff*.	SE(*diff*.)	*z*-value	*p*-value
**Question vs Statement**				
Drug use1-Q1—Drug use1-S1	-7.3	4.2	-1.75	0.079
Drug use1-Q2—Drug use1-S2	3.8	4.3	0.88	0.377
Drug use1-Q3—Drug use1-S3	-9.1	4.6	-1.98	0.048
Drug use1-Q4—Drug use1-S4	3.9	3.9	1.00	0.317
**Factual vs Judgmental**				
Covid-F1—Covid-J1	2.3	4.0	0.56	0.575
Covid-F2—Covid-J2	1.2	4.0	0.29	0.773
Covid-F3—Covid-J3	-1.2	4.1	-0.29	0.772
Covid-F4—Covid-J4	-4.9	4.0	-1.21	0.225

### Analysis of the control question

Table 4 presents the results of the analysis of the control question. For this analysis, the data of the two format conditions of Study I and II were pooled. The percentages of incorrect answers on the ECWM control questions show a clear negative correlation with the length of the number sequence randomizer. Specifically, error rates in Study I using a 15-number sequence (with a probability of 1 of the unrelated question) are significantly higher than those in Study II using a 10-number sequence (with a probability of 1 of the unrelated question) (*X*^2^ = 4.14, *df* = 1, *p* = 0.021). Additionally, the highest error rate of 13.2% was found in Study I using a 15-number sequence, and the lowest error rate of 6.9% was found in the Study III using a 5-number sequence (*X*^2^ = 25.09, *df* = 1, *p* < 0.001). This result strongly suggests that part of the errors was due to overlooking the memorized number in the second sequence. These kind of errors may also (partly) explain the significant *G*^2^ statistics in Study I. The error rates of 3.8% and 3.5% in the DQ condition of Studies II and III suggest some form survey fatigue/rush among the respondents, but they also suggest that part of the errors in the ECWM are due to survey fatigue/rush. For the ECWM condition of Study III this would mean that about 3.5% of the errors are attributable survey fatigue, and about 3.4% to some kind of misinterpretation of the ECWM instructions.

**Table 4:** Analysis of control question.

	error rate in %		parameter estimates	
	DQ	ECWM	*logtime*	*difficulty*
Study I (15-number sequence)	–	13.2	−0.001	0.125
Study II (10-number sequence)	3.8	10.7	−0.148	0.222**
Study III (5-number sequence)	3.5	6.9	0.083	0.458***

* *p* <.05,

** *p* <.01,

*** *p* <.001.

In an effort to explain the incorrect answers to the control question in the ECWM conditions, we performed a series of standard logistic regressions with the standardized scores of the variables *logtime* (the logarithm of the time to complete the survey) and *difficulty* (the perceived difficulty of the ECWM instructions) as predictors. To facilitate comparison of the effects sizes, both predictors were standardized. There are no significant effects of *logtime*, but the effects of *difficulty* show a clear correlation with the length of the number sequence randomizer. The parameter estimates show that the probability of a response error increases with the perceived difficulty of the instructions, and that this effect becomes stronger as the length of the number sequence randomizer decreases. This result strongly suggests that the response errors are due to a mixture of overlooking the memorized numbers and misunderstanding the instructions, and that probability of the former occurring decreases as the length of the number sequence decreases.

A reviewer pointed out that there may be a negative association between education levels of the respondents and the error rates on the control question. In fact, in studies [19, 22] lower-educated respondents were found to produce more errors in the (E)CWM condition. We therefore conducted standard logistic regression analyses with the error rate as dependent variable and education as a nominal covariate (with 5 categories, including the level “other”) and compared the fit of the model with that of the intercept-only model using the difference in *G*^2^ statistics Δ*G*^2^. The results showed no significant effect of education in Study I (Δ*G*^2^ = 5.51, *df* = 4, *p* = 0.239), and Study III (Δ*G*^2^ = 2.90, *df* = 4, *p* = 0.576). In Study II the effect of education was significant (Δ*G*^2^ = 10.03, *df* = 4, *p* = 0.040), with undergraduate and postgraduate respondents producing significantly less errors than respondents with GCSE or below.

## Discussion

This paper evaluated the performance of the ECWM in combination with a number sequence randomizer. The ECWM distinguishes itself from other randomized response techniques by the use of the two neutral response options “I have TWO ‘Yes’ or TWO ‘No’ answers” and “I have only ONE ‘Yes’ answer” of the CWM, and by the use of two sub-samples with complementary probabilities of answering “Yes’’ to the unrelated question. This latter property allows for a goodness-of-fit test. The number sequence randomizer is proposed as an alternative for the birthday question randomizer, that is commonly used in other applications of the CWM and ECWM.

The (E)CWM with a birthday randomizer has been investigated in numerous validation studies. The (E)CWM has been shown to provide higher levels of comprehension and perceived privacy protection [19] than other randomized response techniques. “Weak” validation studies, i.e. studies comparing the prevalence estimates obtained through the (E)CWM and DQ for sensitive attributes with unknown prevalence, have provided evidence for both the “more-is-better” criterion for socially undesirable attributes [15, 17, 25, 27, 29, 40], and the “less-is-better” criterion for socially desirable attributes [23]. Moreover, in “strong” validation studies, i.e., studies of a sensitive attribute with a known prevalence, the CWM yielded estimates that were close to the known prevalence [41, 42]. However, some validation studies of the CWM yielded results that were not in agreement with the “more-is-better” criterion [18, 42, 43]. A potential explanation of these latter results is the experimental character of the sensitive attributes, which either had a zero prevalence [44] or were experimentally induced [20, 22].

Although the validation studies with “non-experimental” sensitive attributes seem to support the validity of the (E)CWM, random responding has been identified as an alternative explanation for confirmation of the “more/less-is-better” criteria [44–46]. Random responding may occur due to insufficient comprehension of the instructions or inattentiveness or carelessness, and bias the prevalence estimates toward 50% for both socially desirable and undesirable attributes. In studies by [22, 47, 48], 2 to 19% of the respondents indeed admitted to answered the questions randomly. A study by [28] however strongly suggests that, while random responding cannot be ruled out completely, it only plays a minor role in conformations of the “more/less-is-better” criteria.

In general, the above-mentioned validation studies provided evidence for the validity of the(E)CWM with a birthday randomizer. The central question of the current paper is how the ECWM with the number sequence randomizer relates to the findings of these validation studies. It has already been argued that number sequence randomizer has some theoretical advantages over the birthday randomizer, the main two being the elimination of potential biases due to informed self-protection and due to a non-uniform distribution of birthdays over the days or months of the year. Another advantage that has not been mentioned yet is that the number sequence randomizer is easier to implement when multiple sensitive questions are asked, as was the case in the three studies we presented in this paper. It is no problem to present the respondent with five different sets of number sequences, but it is much more difficult to come up with five different birthday questions. One option is to use the same person’s birthday but to select different periods, e.g., the months January and February for the first question, the months March and April for the second, etc. Problems with this approach are that it may decrease perceived privacy protection, especially if the person’s birthday could be known to the researcher, and it excludes a multivariate analysis of the questions because the probabilities to answer “Yes” to the birthday questions are no longer independent. This dependence issue is solved by using the birthdays of different persons, like the father’s and the mother’s birthday, but what persons to select for the remaining three questions? Finally, the number sequence randomizer is also better suited for “strong” validation studies because it allows the randomization probabilities to be set to either 0 or 1, thus eliminating uncertainty in the prevalence estimates due randomization. Obviously, setting the randomization probabilities to 1 and/or 0 is not possible with birthday questions.

Our study yielded some promising empirical results that are inline with the evaluation studies of the (E)CWM with birthday randomizer. Practically all prevalence estimates of the ECWM were in line with the “more-is-better” criterion; the ECWM yielded higher prevalence estimates for the sensitive questions and similar prevalence estimates for non-sensitive questions. The latter result can also be taken as an indication that random responding has not played a major role, because in that case the ECWM would have yielded higher prevalence estimates than DQ. In general, the goodness-of-fit tests showed a satisfactory fit. Three of the six significant *G*^2^ statistics only exceeded the critical value of 3.84 by a small margin, and the two of the three largest *G*^2^ statistics occurred on the first question and in Study I with the 15 number sequences. These results suggest that respondents may not have fully understood the instructions on the first question, and that the 15-number sequences are more prone to response errors than the shorter number sequences. This last explanation seems to be confirmed by the analysis of the control question, which suggests that shorter number sequence results in less response errors. The non-zero error rates on the control question in the DQ conditions suggests that there is some degree of survey fatigue/rushed completion, which may have also contributed to the *G*^2^’s of the significant goodness-of-fit tests in the ECWM conditions.

Somewhat to our surprise we found no differential effects for judgmental versus factual question formats. A potential explanation for this finding is that aside of the wording of the questions, other factors like the appropriateness and context of the question also play an important role in the willingness of the respondent to answer honestly. In a study on the effects of the wording of the sensitive questions on the willingness to give honest answer, [49] found mixed results.

The statistical contribution of this paper concerns the development of a maximum likelihood estimator for the prevalence of the sensitive characteristic, and its extension to a binary logistic regression model that can be used to investigate the effects of covariates on the probability of having the sensitive characteristic. We have illustrated its use by performing a logistic regression with sub-sample membership as covariate, and shown that the results are equivalent to conducting the goodness-of-fit test.

The current study has some limitations. Firstly, since the validation only involved comparisons of the ECWM and DQ conditions, we have no empirical evidence for the superiority of the number sequence randomizer over the birthday randomizer since our study lacked a condition with the birthday randomizer. A second limitation of our study is that as yet we do not have a satisfactory explanation for the misfit of some of the ECWM questions, except for the conjecture that the misfit on the first questions may be due to insufficient comprehension of the instructions. However, a misfit for ECWM questions is not specific to the number sequence randomizer. For example, [28] observed a misfit on three of the eight ECWM questions using the birthday randomizer. Finally, our observation that the error rates on the control question increased with the length of the number sequences led us to the conclusion that the longer sequences are more prone to errors than the shorter ones. Due to the confounding of randomization probabilities and the studies, with Study III having the lowest error rates and a randomization probability of 0 and Studies I and II having the highest error rates and randomization probabilities of 1, it cannot be ruled out that the randomization probabilities had an effect on the error rates. To avoid such confounding, we have set the randomization probability of the control question to 0 in one sub-sample, and to 1 in the other sub-sample in new surveys we are conducting.

## Conclusion

In summary, the ECWM with the 5-number sequence randomizer exhibited a good performance in estimating the prevalence of sensitive issues. Its design offers a good combination of features that collectively ensure, as much as possible, that the data it generates are valid and reliable. The ECWM with number sequence randomizer reduces the risk of response biases since it does not offer an obvious strategy for self-protective responses, while its use of two sub-samples stills allows testing for other forms of response bias. With its relatively simple instructions and its short length, the 5-number sequence randomizer also reduces the risk of response errors. Furthermore, it is also well suited for the anticipated large scale applications, for instance, international sport events. As a tool for WADA, a sequence of questions with incremental sensitivity is recommended, along with a control question to provide an indication of the degree to which the respondents understood the instructions.

## Supporting information

S1 Fig Power curves of the crosswise model. (TIF)

S2 Fig Allocation of respondents to experimental conditions. (TIF)

S1 Appendix Background characteristics of the participants in the three studies. (PDF)

S1 Table Summary of the project design in Studies I, II and III. (PDF)
