# Supplementary figures and images for: Refinement of the extended crosswise model with a number sequence randomizer: Evidence from three different studies in the UK
Source: PLoS One. 2022 Dec 30;17(12):e0279741. doi: 10.1371/journal.pone.0279741 (PMC9803288; doi:10.1371/journal.pone.0279741)

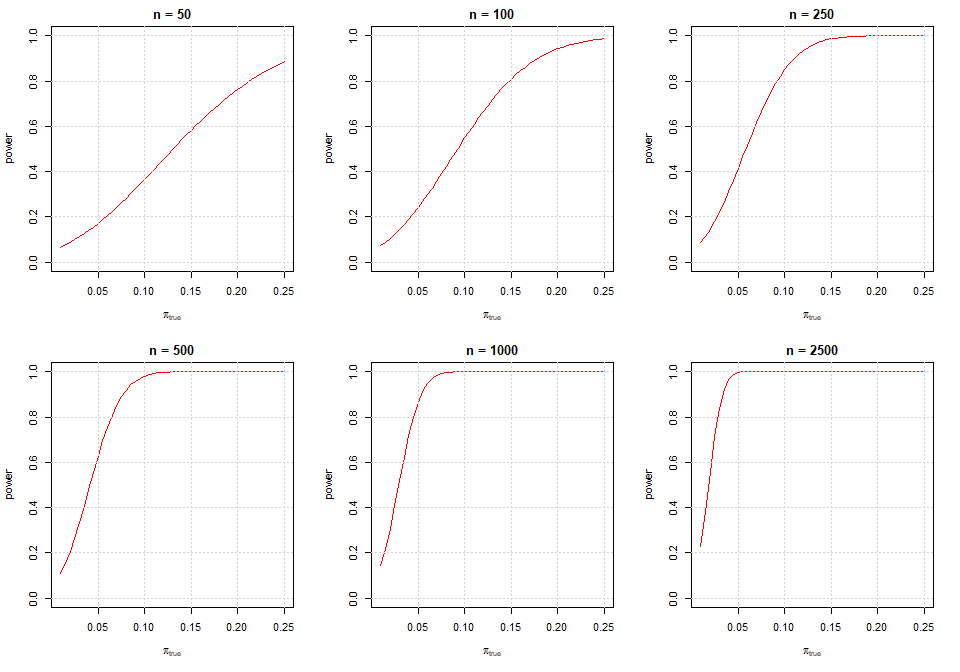

Supplement: S1 Fig — (TIF) [file pone.0279741.s001.tif]

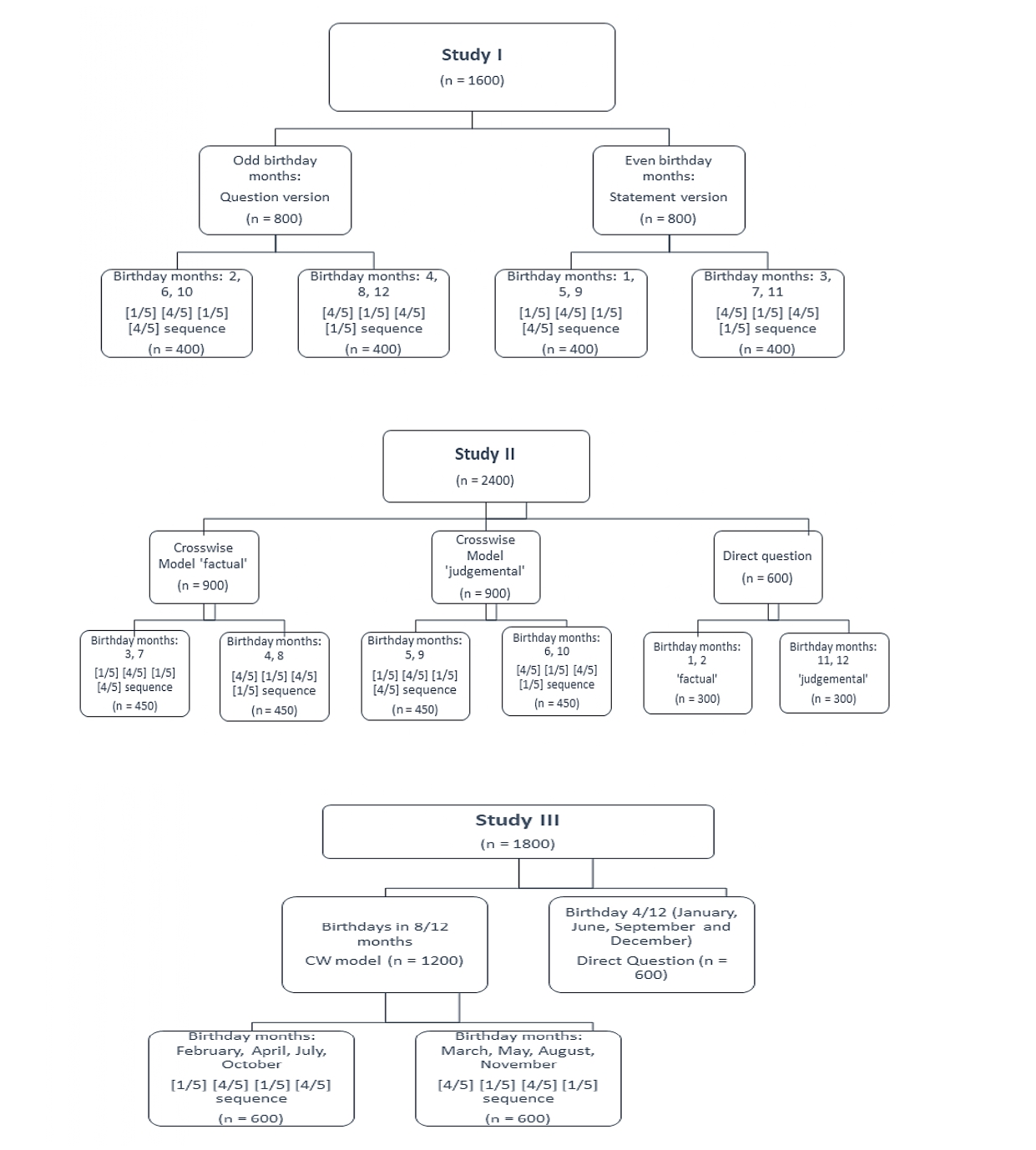

Supplement: S2 Fig — (TIF) [file pone.0279741.s002.tif]
