# Supplementary material for: Refinement of the extended crosswise model with a number sequence randomizer: Evidence from three different studies in the UK
Source: PLoS One. 2022 Dec 30;17(12):e0279741. doi: 10.1371/journal.pone.0279741 (PMC9803288; doi:10.1371/journal.pone.0279741)
Supplement: S1 Appendix — (PDF) [file pone.0279741.s003.pdf]

S1 Appendix

Background Statistics of respondents

The data of Study II: compliance with Covid-19 regulations, were collected online via Prolific Academic platform on May 22nd, 2020, ten days after the UK lockdown measures were announced. Table 1 presents summary statistics of demographic characteristics of the respondents who were in the UK during the lockdown period for the total sample ($n = 2402$) and for the DQ ($n = 781$) and the ECWM ($n = 1621$) conditions separately. The last column shows the p-values of the ANOVAs (after verifying its assumptions, which were met) testing the mean differences of the continuous variables (age, time to complete survey) between the DQ and ECWM conditions, whereas for the categorical variables (gender, sport activities, levels of education) the Chi-square test of independence is used. It shows no significant differences, except for the time spent to complete the survey; it took respondents in the ECWM condition significantly longer to fill out the survey than respondents in the DQ condition.

Table 1. Summary statistics of Study II: Covid-19 regulations

	**Total**	**DQ**	**ECWM**	
**Age (years)**				
Mean	38.2	38.2	38.2	t = -0.01, p-value = 0.99
SD	13.0	12.8	13.0	
**Time complete survey (minutes)**				
Mean	5.0	4.4	5.3	t = 5.4, p-value< 0.001
SD	4.0	5.8	2.7	
**Gender**				
Female	1529	493	1036	$\chi^2(2) = 0.25$, p-value=0.87
Male	869	287	582	
Other	4	1	3	
**Sport Activities**				
Regular exercise	996	325	671	$\chi^2(4) = 0.85$, p-value=0.93
Occasional exercise	894	295	599	
Not involved	381	122	259	
Competitive; local/university club	85	24	61	
Other activities	46	15	31	
**Level of Education**				
Undergraduate	864	282	582	$\chi^2(6) = 3.61$, p-value=0.73
A-levels/B-Tech/College	589	193	396	
Postgraduate	450	146	304	
GCSE or below	285	95	190	
Other	214	65	149	

The study of controlled substances was carried out in two different rounds. In the first round (Study I), which took place in February, 2020, the sensitive questions were posed in two different ways: as a statement or as a query. In contrast to the Covid-19 study, the time to complete the survey is not significantly different. With the exception of the age, the respondents in the ECWM condition with the Question format ($n = 734$) did not differ significantly from the respondents in the ECWM condition with the Statement format ($n = 771$) with respect to the socio-demographic characteristics (Table 2). The DQ was not employed in this study.

Table 2. Summary statistics of Study I: controlled substances

	Total	ECWM-Question	ECWM-Statement	
**Age (years)**				
Mean	28.4	28.9	28	t = - 2.21, p-value = 0.03
SD	7.1	7	7.2	
**Time complete survey(minutes)**				
Mean	4.7	4.7	4.7	t = -0.22, p-value= 0.82
SD	3.3	3.2	3.4	
**Gender**				
Female	732	369	363	$\chi^2(2) = 2.19$, p-value=0.33
Male	763	359	404	
Other	10	6	4	
**Sport Activities**				
Regular exercise	577	273	304	$\chi^2(4) = 2.18$, p-value=0.70
Occasional exercise	565	283	282	
Not involved	207	106	101	
Competitive; local/university	67	29	38	
Other activities	89	43	46	
**Level of Education**				
Undergraduate	414	215	199	$\chi^2(6) = 7.3$, p-value=0.29
Postgraduate	340	165	175	
A-levels/B-Tech/College	285	142	143	
Year 2 (university)	163	73	90	
Other	303	139	164	

Table 3 presents summary statistics of demographic characteristics of Study III for the total sample ($n = 1801$) and for the DQ ($n = 590$) and the ECWM ($n = 1211$) conditions separately. The last column shows that in spite of the random assignment to the conditions there are some differences between the conditions with respect to sport activities and education. Additionally, the average age in Study III was higher than Study I.

Table 3. Summary statistics of Study III: controlled substances

	Total	DQ	ECWM	
**Age (years)**				
Mean	34.0	34.2	33.8	t = - 0.77, p-value = 0.44
SD	10.3	10.2	10.3	
**Time complete survey (minutes)**				
Mean	5.9	5.8	5.9	t = 0.89, p-value=0.37
SD	3.7	2.7	4.1	
**Gender**				
Female	1129	365	764	$\chi^2(2) = 0.31$, p-value=0.85
Male	664	222	442	
Other	8	3	5	
**Sport Activities**				
Regular exercise	737	260	477	$\chi^2(4) = 10.90$, p-value=0.03
Occasional exercise	653	206	447	
Not involved	230	61	169	
Competitive; local/university club	115	34	81	
Other activities	66	29	37	
**Level of Education**				
Undergraduate	637	206	431	$\chi^2(6) = 13.59$, p-value=0.03
A-levels/B-Tech/College	467	129	338	
Postgraduate	305	117	188	
GCSE or below	189	67	122	
Other	203	71	132	
