# Supplementary material for: Refinement of the extended crosswise model with a number sequence randomizer: Evidence from three different studies in the UK
Source: PLoS One. 2022 Dec 30;17(12):e0279741. doi: 10.1371/journal.pone.0279741 (PMC9803288; doi:10.1371/journal.pone.0279741)
Supplement: S1 Table — (PDF) [file pone.0279741.s004.pdf]

S1 Table

Summary of the project design in Studies I, II and III

	Study I	Study II	Study III
Target Sample Size	1,600	2,400	1,800
Sensitive Behavior	Controlled substance use	Covid-19 lockdown rule compliance	Controlled substance use
Number Sequence	15 two-digit numbers	10 two-digit numbers	5 two-digit numbers
Number of questions	4 + 1 control question	4 + 1 control question	4 + 1 control question
Control Question	Set to p = 1.0	Set to p = 1.0	Set to p = 0.0
Direct Question	no	yes	yes
Areas of Interest	1. Unique number sequences for independence of the questions<br><br>2. Question vs Statement format	1. Impact of reducing number sequence for the unrelated question<br><br>2. Factual vs Judgmental format<br><br>3. Comparison to direct question	1. Impact of reducing number sequence for the unrelated question<br><br>2. Comparison to direct questions<br><br>3. Impact of setting control question at p = 0.0
Feedback (absolute, subjective assessment of format)	Yes<br><br>(bipolar; very difficult – very easy)	Yes<br><br>(bipolar; very difficult – very easy)	Yes<br><br>(bipolar; very difficult – very easy)
